# Effects of diamond nanoparticle surface composition and the sp^3^/sp^2^ carbon ratio on tumor proangiogenic potential in vitro

**DOI:** 10.1038/s41598-025-14943-8

**Published:** 2025-08-13

**Authors:** Katarzyna Zawadzka, Barbara Wójcik, Sławomir Jaworski, Agnieszka Ostrowska, Artur Małolepszy, Marta Mazurkiewicz-Pawlicka, Marcin Pisarek, Wiktoria Frączek, Mateusz Wierzbicki

**Affiliations:** 1 Department of Nanobiotechnology, Institute of Biology, Warsaw University of Life Sciences, Bldg. 23, rm. 133, 8 Ciszewskiego Str, Warsaw, 02-786 Poland; 2 Faculty of Chemical and Process Engineering, Warsaw University of Technology, Warsaw, 00-654 Poland; 3 Institute of Physical Chemistry, Polish Academy of Sciences, Warsaw, 01-224 Poland

**Keywords:** Diamond nanoparticles, Tumor, Angiogenesis, sp^3^ carbon, Endothelial cells, Tumor microenvironment, Tumour angiogenesis, Nanoparticles

## Abstract

The high proangiogenic potential of tumors is often associated with poor prognosis due to increased invasiveness and malignancy. Diamond nanoparticles (NDs) are considered a promising anti-tumor agent with anti-angiogenic properties; however, their activity is strictly connected with their physicochemical parameters and surface chemical composition. One of the main factors characterizing detonation NDs is the sp^2^ surface carbon content, which can determine the character of nanoparticle–cell interaction. The primary objective of this study was to investigate the influence of different types of NDs on the proangiogenic potential of tumor cells distinguished by phenotype and invasiveness. NDs with a high sp^3^/sp^2^ carbon ratio decreased the levels of crucial proangiogenic proteins secreted by T98G and HepG2 cells (IL-6, IL-8, ANG, TIMPs, and ANGPTs). Moreover, a lower sp^2^ carbon content on the surface of NDs reduced oxidative stress in both mesenchymal T98G and epithelial HepG2 tumor cells and affected the NF-κB activation state in a cell-specific manner. Modified NDs, by affecting the tumor cell secretome composition, indirectly inhibited endothelial cell migration and tube formation, demonstrating high cell type specificity. Taken together, the results presented here indicate the significance of the surface parameters of NDs in the indirect regulation of cellular signaling and the microenvironment.

## Introduction

Angiogenesis is one of the crucial processes determining tumor progression. Within the tumor microenvironment, the increased expression of proangiogenic factors prompts rapid and disorganized expansion of the microvascular network, thus contributing to faster tumor growth and facilitating metastasis^1^.

Despite the existence of many developed treatment strategies, tumors with stronger angiogenic potential constantly occupy leading positions in cancer-related mortality statistics^2^. To date, numerous antiangiogenic therapeutics have demonstrated promising results in both preclinical models and clinical trials, which has led to the approval of some of them for systemic or side treatment of tumors such as metastatic breast cancer, unresectable hepatocellular carcinoma, and glioblastoma, although it is still a major challenge for the effects achieved in in vitro models to be reflected in long-term therapy^3–6^.

Diamond nanoparticles (NDs) exhibit many features, indicating their potential for biomedical applications. Thus far, the biocompatibility of detonation NDs has been repeatedly proven in in vitro and preclinical animal models^7,8^. NDs are effectively internalized into cells and can be detected inside them for up to several generations^9^, which is one of the reasons they have revealed potential as active drug carriers improving their uptake, distribution, and effectiveness^10^. An additional advantage they have is in downregulating some of the key proangiogenic proteins, which indicates that NDs could simultaneously aim for multiple therapeutic targets^11^. NDs exhibit cell type-specific interactions, including time- and dose-dependent toxicity toward endothelial cells, as shown in other studies^12^.

While the production of NDs by detonation enables efficient and cost-effective large-scale manufacturing, the resulting nanoparticles often contain a higher content of surface graphitic layer^13^. Further preparation of nanoparticles requires purification; therefore, the feature that differentiates functionalized NDs is the shift in the sp^3^/sp^2^ carbon ratio^13^. Surface modification affects their physicochemical properties, such as surface charge and colloidal stability, and as a consequence may influence the specificity of ND interactions with cells^14^. Currently, there are only a few reports on differences caused by ND surface composition in biological models^15^. Therefore, a deeper understanding of the differences in the cellular response to various forms of NDs is essential for designing ND-based biomedical solutions.

The aim of this study was to evaluate the significance of surface chemistry and the sp^3^/sp^2^ carbon ratio, which is a prominent differentiator among detonation-produced NDs in their role in regulating the tumor microenvironment in in vitro coculture models.

## Materials and methods

### Characterization of diamond nanoparticles

NDs produced via the detonation method were purchased from US Research Nanomaterials (Houston, TX, USA). Raw, unmodified NDs were designated as ND-R, whereas surface-purified nanoparticles were defined as ND-M1, ND-M2, and ND-M3. According to the producer, ND-M1, ND-M2, and ND-M3 were obtained through hydroxylation, amination, and carboxylation processes, respectively. Prior to use, the nanopowders were dissolved in ultrapure Milli-Q water to prepare 1000 mg/L solutions, then sonicated for 5 min at 500 W and 20 kHz with 30 s intervals in a VC 505 Ultrasonic Liquid Processor with a cup horn (Sonics & Materials, Newton, CT, USA). Subsequently, the stock solutions were diluted using ultrapure Milli-Q water to 50, 100, 200 and 500 mg/L for further use.

The zeta potential describing the surface charge and hydrocolloidal stability in the pH range of 3 to 9 was ascertained using the Smoluchowski approximation, and the hydrodynamic diameter was assessed by dynamic light scattering (DLS). Measurements were performed in triplicate using a Zeta Sizer Nano-ZS90 analyzer (Malvern, Worcestershire, UK) after stabilization for 2 min at 25 °C. The pH of the hydrocolloids was measured using an ELMETRON CP-411 pH meter (Elmetron, Zabrze, Poland). The morphology of the nanoparticles was evaluated by transmission electron microscopy (TEM). Samples of nanoparticle suspensions at 20 mg/L were deposited on copper grids and allowed to dry. Images were taken with a JEM-1220 transmission electron microscope (JEOL, Tokyo, Japan) at 80 KeV with a Morada 11-megapixel camera (Olympus Soft Imaging Solutions, Münster, Germany). Nanoparticle size distribution was determined from 200 individual measurements obtained from three TEM images using ImageJ software^16^. The resulting data were used to generate the size histograms.The chemical composition of the ND powders was characterized by Fourier transform infrared (FT-IR), Raman, and X-ray photoelectron spectroscopy (XPS).

Raman spectra were obtained with an inVia Renishaw microscope with a 514 nm laser (Renishaw, Gloucestershire, United Kingdom). The following conditions were used for the measurements: lens, 50x; each measurement site was exposed five times for 5 s with a 5% laser intensity. The second method for investigating the ND surface structure was FT-IR spectroscopy using a Nicolet iS10 (Thermo Fisher Scientific, Waltham, MA, USA) spectrometer. The results were established taking into consideration the background for “dry air.” Pellets for measurements were obtained by mixing the samples with KBr at a ratio of 1/300 mg, followed by compression at 7 MPa cm^− 2^. The spectra were collected in the range of 400–4000 cm^− 1^.

XPS photoelectron spectroscopy was used to analyze the chemical composition of the surfaces of the modified and unmodified NDs and to determine the chemical state of the elements identified during the measurements using a Microlab350 spectrometer (Thermo Fisher Scientific). The measurements were carried out using an X-ray excitation source with an energy of 1486.6 eV (Al anode, non-monochromatic). High-resolution XPS spectra were recorded with a step of 0.1 eV and pass energy of 40 eV at a pressure of 10 − 9 mbar, which were then processed using Thermo Avantage software version 5.9911. All recorded high resolution XPS spectra were corrected for C-C bonds (sp^2^ at an energy of 284.5 eV.

### Cell lines

Human glioblastoma cell lines U-118 MG and T98G, hepatocellular carcinoma cells HepG2, and two breast cancer cell lines MDA-MB-231 (triple-negative breast cancer) and MCF7 (ER+/PR+) were purchased from the American Type Culture Collection (Manassas, VA, USA). The cells were cultured in accordance with the supplier’s instructions in EMEM (ATCC) supplemented with 10% fetal bovine serum (FBS) and 1% penicillin/streptomycin (1000 µg/mL) at 37 °C in a humidified atmosphere with 5% $\:{\text{C}\text{O}}_{2}$ (Gibco, Thermo Fisher Scientific). Only the U-118 MG cell line was maintained in DMEM with identical supplements. Cells were used in experiments no later than the tenth passage.

Human primary endothelial cells (HUVECs) were maintained in endothelial cell growth medium (ECGM) supplemented with SupplementMix. Cells and dedicated culture media were purchased from Promocell (Heidelberg, Germany). The HUVECs used for the tests were at the fifth passage at the latest.

### Direct cytotoxicity of NDs

The metabolic activity of the tumor cell lines was evaluated by XTT assay (CyQuant, Thermo Fisher Scientific). HepG2, U-118 MG, T98G, MCF-7, and MDA-MB-231 cells were seeded in 96-well plates at a concentration of 1×$\:{10}^{4}$ cells per well and then incubated at 37 °C under standard conditions. Twenty-four hours after seeding, ND-R, ND-M1, ND-M2, and ND-M3 hydrocolloid stock solutions at concentrations of 50, 100, 200, 500, and 1000 mg/L were added to the cells in a volume equal to 10% of the total fresh medium, resulting in final concentrations of 5, 10, 20, 50, and 100 mg/L, respectively. After subsequent 24 h, the cell culture medium was replaced with fresh medium. Thereafter, 70 µL of XTT reagent was added to each well. Plates were incubated for 3 h at 37 ˚C, protected from light. Absorbance was measured at 450 nm with a reference at 600 nm using a Tecan Infinite 200 microplate reader (Tecan Group Ltd., Männedorf, Switzerland). Cell viability was expressed as a percentage of the untreated control group with cell-free blanks included. The assay was conducted at least in triplicate and with three independent repetitions.

Cell membrane integrity was examined using the Invitrogen CyQUANT LDH Cytotoxicity Assay (Thermo Fisher Scientific). Cells were seeded in the same manner as described above. After 24 h, the cell culture medium was aspirated and replaced with fresh medium with 2% FBS to minimize the background effect in absorbance measurements caused by higher concentrations of FBS. The medium contained NDs diluted to 5, 10, 20, 50, and 100 mg/L as 10% of the volume in each well. After 24 h of incubation under standard conditions, the LDH assay was performed. In accordance with the manufacturer’s instructions, cells in the positive control group were lysed for 45 min, the plates were subsequently centrifuged for 5 min at 200×g, and 50 µL of the cell culture medium was transferred to a new testing plate. Thereafter, 50 µL of LDH reaction mixture was added to each well. The plates were incubated and protected from light, with constant agitation for 30 min at room temperature. The absorbance was measured at 490 nm (with a reference at 680 nm) after the reaction had been stopped by adding Stop Buffer to each well and mixing thoroughly. The results are presented as a percentage of the positive control (lysed cells). Spontaneous LDH leakage from untreated cells constituted a negative control. The assay was performed at least in triplicate and repeated three times.

The morphological changes in the tumor cells were examined using an inverted phase-contrast CKX 41 Olympus microscope (Olympus, Tokyo, Japan). Images of living cells were taken at 20x magnification 24 h after introducing nanoparticle solutions at concentrations of 20 mg/L and 50 mg/L using a ProgRes c12 camera (Jenoptik, Jena, Germany).

### Characterization of tumor-conditioned cell culture medium (CM)

To obtain tumor-conditioned cell culture medium (CM) MDA-MB-231, MCF-7, and U-118 MG cells were seeded at $\:{2\times\:10}^{5}$, HepG2 at $\:{3\times\:10}^{5}$, and T98G at $\:{1.5\times\:10}^{5}$ cells per well on six-well plates, which led to 70% confluence after 24 h of cultivation. At that point, hydrocolloids of diamond nanoparticles at concentrations of 20 and 50 mg/L were introduced to tumor cells in serum-free culture medium (ATCC). After 24 h of incubation, the medium was harvested, centrifuged at 5000 rpm at 4 °C, and stored at -80 °C for further use.

The proangiogenic protein profile of CM was examined using the Human Angiogenesis Antibody Array (ab193655, Abcam, Cambridge, UK), which was utilized to determine the levels of 43 proangiogenic proteins in the selected cell lines. The complete array map and exact protein localizations are provided in Supplementary Table S1, Supplementary Table S2, and Supplementary Fig. S7. The analyzed CMs were derived from T98G and HepG2 cells treated with ND-R and modified ND-M1, ND-M2, and ND-M3 hydrocolloids at a concentration of 50 mg/L. The samples were undiluted and added in a volume of 1 mL per membrane. Chemiluminescence signals were detected with the Azure Biosystem C400 (Azure, Dublin, CA, USA), followed by pixel density analysis with the “Protein Analyzer” plugin from ImageJ^16^. The results were normalized to positive and negative controls and are expressed as the average of two replicates.

### Characterization of the proangiogenic potential of tumor-conditioned cell culture medium (CM)

The proangiogenic potential of CM was determined by the HUVEC tube formation assay and HUVEC migration in an indirect coculture model.HUVECs used for the tube formation assay were seeded on 12-well plates at a concentration of $\:{1\times\:10}^{5}$ cells per well 48 h prior to the test. After 24 h, the cell culture medium was replaced with fresh basal ECGM (without supplements) with CM at a 1:1 ratio. HUVECs were incubated with CM for 24 h, trypsinized, and seeded in a µ-Slide 15 Well 3D (Ibidi, GmbH, Gräfelfing, Germany) at a concentration of $\:{1.2\times\:10}^{4}$ cells/well in 50 µL of total medium volume containing basal ECGM 1:1 with CM. Fresh EMEM with basal ECGM was used as a negative control. Before adding the cells, the wells were filled with 10 µL of Geltrex LDEV-Free Reduced Growth Factor Basement Membrane Matrix (Thermo Fisher Scientific), which was subsequently polymerized by 1 h incubation at 37 °C. Tubes were observed and photographed under an inverted phase-contrast CKX 41 Olympus microscope (Olympus) with a ProgRes c12 camera (Jenoptik) in the eighth h of incubation at 37 °C. The total tube length and total number of junctions were analyzed using the “angiogenesis analyzer” macro in ImageJ^18^.

The results were obtained by summing the values acquired from two independent experiments performed at least in triplicate. A diagram illustrating the analysis of total tube length and number of junctions in the field of view (FOV) from ImageJ is presented in the Supplementary Information (see Supplementary Fig. S6).

HUVEC migration in CM was assessed by a cell exclusion zone assay. HUVECs were seeded in the chambers of silicon inserts (Ibidi) attached to 24-well plates. A total of 1 × 10⁴ cells per chamber were allowed to cover the available space for 24 h in full ECGM. Afterwards, the inserts were gently removed, and the cell culture medium was exchanged for ECGM and CM combined 1:1. Images were captured within 1 h after treatment and 24 h after treatment using a Leica DMi8 microscope equipped with a Leica MC 190 HD camera (Leica Microsystem, Wetzlar, Germany). The images were analyzed using the “wound healing tool” macro in ImageJ^19^. The results were obtained by pooling the measurements acquired from two independent experiments, each performed in triplicate, and are presented as the percentage of area unoccupied by HUVECs after incubation for 24 h relative to measurements taken within 1 h of removing the inserts and the treatment of cells’.

### Intracellular ROS level detection

Total reactive oxygen species (ROS) production in selected tumor cell lines was evaluated with CM-H2DCFDA (General Oxidative Stress Indicator, Invitrogen, Thermo Fisher Scientific). T98G and HepG2 cells were seeded onto µ-Slide eight-well chambered coverslips (Ibidi) at a concentration of 3 × 10^4^ cells/well. After 24 h, ND-R, ND-M2, and ND-M3 hydrocolloids were introduced to the cells at a concentration of 50 mg/L in EMEM without FBS. Following 24 h of incubation with nanoparticles, the medium was removed, and the cells were washed twice with Hank’s Balanced Salt Solution (HBSS, Thermo Fisher Scientific). Subsequently, CM-H2DCFDA suspended in HBSS at a concentration of 5 µM was added, followed by a 20 min incubation at 37 °C. The cells were then washed twice again with HBSS, and the nuclei were stained with 5 µg/mL Hoechst 33,342 (Invitrogen, Thermo Fisher Scientific) in culture medium (15 min; 37 °C). For visualization, the cells were washed three times with HBSS and placed in culture medium buffered with 25 µM HEPES. Images were taken under an FV-1000 confocal microscope (Olympus Corporation, Tokyo, Japan). The experiment was conducted twice, with four replicates for each treatment. ROS production was quantified by computing the pixel density from green fluorescence (CM-H2DCFDA) of two regions for each image to the number of nuclei in each region. Cell counting and fluorescence intensity measurements were performed using Fiji software^16^.

### NF-κB activity

NF-κB p65 subunit activity was measured with a TransAM NF-κB kit (Active Motif, Carlsbad, CA, USA), which is an ELISA-based DNA-binding assay. Nuclear extracts obtained from T98G and HepG2 cells treated with ND-R and ND-M3 for 24 h at a concentration of 50 mg/L were used for analyses. The nuclear fraction was isolated using an NE-PER™ Nuclear and Cytoplasmic Extraction Reagents kit (Thermo Scientific). Analyses were performed in two independent repetitions, each with three replicates per group containing 20 µg of protein sample. After applying the extracts to the plate, incubations with the primary antibody and the HRP-conjugated secondary antibody were carried out according to the manufacturer’s recommendations. The results were obtained by reading the absorbance at 450 nm and are presented as the relative difference from the control group.

Whole-cell lysates for western blot analysis were prepared from one 75 cm^2^ culture flask per group. The cells were treated in the same manner as described above, harvested by scraping in HBSS, and then centrifuged (300 × g, 5 min.). The resulting pellets were suspended in radio-immunoprecipitation assay (RIPA) buffer with Halt Protease & Phosphatase Inhibitor Cocktail and 5 mM EDTA (Thermo Fisher Scientific). The samples were sonicated for 1 min at 500 W and 20 kHz with 5 s intervals, followed by incubation for 40 min with vortexing every 10 min. Afterwards, cell extracts were centrifuged for 30 min at 12,000 × g, and the supernatants were transferred to fresh tubes. Every step of the procedure was performed at 4 °C/on ice. The total protein concentration was determined using a Pierce BCA Protein Assay Kit (Thermo Fisher).

The cell lysates were diluted to equal concentrations and volumes and denatured with Laemmli Sample Buffer with 2-mercaptoethanol (Bio-rad, California, USA) for 5 min at 95 °C. Polyacrylamide gels were prepared using a TGX Stain-Free FastCast Acrylamide Kit (7.5%, Bio-Rad). For the loading control of total protein, stain-free gels were activated under UV light for 1 min and then visualized. Subsequently, proteins were transferred to nitrocellulose membranes with a Trans-Blot Turbo Transfer System (Bio-Rad). The membranes were blocked with I-Block Protein-Based Blocking Reagent (Thermo Fisher Scientific) or iBind™ Buffer (Invitrogen, Thermo Fisher Scientific) for phosphoprotein detection. The membranes were incubated with primary antibody (cat. 51–0500, 44-711G Thermo Fisher) overnight with gentle agitation, followed by washing in TBST buffer and a 2 hincubation with secondary antibody (ab97048, Abcam). CDP-Star Substrate (Thermo Fisher Scientific) with Nitro-Block-II Enhancer (Thermo Fisher Scientific) were used for visualization. After phosphorylated protein detection, the membranes were stripped with Restore™ Western Blot Stripping Buffer (Thermo Fisher Scientific) and used for whole p65 analysis. Gels serving as loading controls and western blot membranes were visualized with a C400 AZURE system (Azure Biosystems, Dublin, Ireland). Background subtraction was performed using ImageJ software^16^. Images of the gels and membranes are provided in the Supplementary Information (see Supplementary Fig. S8).

### Cellular membrane potential

To evaluate the influence of diamond nanoparticles on tumor cell membrane potential, T98G and HepG2 cells at 4 × 10^4^ cells per well were seeded in 96-well black plate (Ibidi) 24 h prior to the experiment. Cellular Membrane Potential Assay Kit (Fluorometric-orange; ab176764, Abcam) was used according to manufacturer instructions. Cells were incubated with membrane potential (MP) sensor dye-loading solution. After 30 min, cells were treated with ND-R or ND-M3 nanoparticles at a final concentration of 50 mg/L in EMEM medium without FBS.Fluorescence was measured at Ex/Em = 530/570 nm immediately before treatment and again after 24 h of incubation. Data from two independent experiments (*n* = 5) were pooled for statistical analysis, and results are presented as relative values normalized to the untreated control.

### Lipid peroxidation

Lipid peroxidation analysis with Lipid Peroxidation (MDA) Assay Kit (MAK085, Sigma-Aldrich) was performed using cell lysates from 2 × 106 T98G and HepG2 cells per experimental group. Prior to the assay, cells were cultured in 75 cm² flasks and treated with ND-R or ND-M3 at a final concentration of 50 mg/L in EMEM without FBS for 24 h. All of the used reagents were prepared freshly before the assay. Malondialdehyde (MDA) dilutions for a standard curve were prepared using following concentrations: 2.0, 1.6, 1.2, 0.8, 0.4, and 0.0 nmol/well. MDA concentration in the samples was calculated based on the absorbance standard curve and equation below, according to manufacturer protocol. Samples were analysed in triplicates.

MDA (nmol/mL) = (_A_/_V_) × = C.

where: _A_ = Amount of MDA in Sample (nmole) as determined from the standard curve) _V_ = Sample volume (mL) added into the wells DF = Sample dilution factor C = Concentration of MDA in sample”.

### Bioprinted 3D model

T98G glioblastoma cells and HepG2 hepatocellular carcinoma cells were trypsinized and suspended in cell culture medium (ECGM 1:1 EMEM 0% FBS) at a concentration of $\:1.1\times\:{10}^{7}$/mL in 50 µL mixed with 450 µL of GelMA (Cellink, Gothenburg, Sweden). Droplets with encapsulated tumor cells were bioprinted by extrusion using a BIO X 3D printer (Cellink). After 6 days of cultivation in an eight-well chamber (Ibidi) with cell medium exchanged every 2 days, the cells in the droplets were stained with Cell Tracker DeepRed (Thermo Fisher Scientific), treated with hydrocolloids of ND-R and ND-M3 at 50 mg/L, and incubated for a further 48 h (with three replicates per group). Next, HUVECs were plated around droplets at a concentration of 3 × 10⁴ per well. Coculture cultivation was conducted for the next 5 days. Eventually, the cells were fixed with 4% paraformaldehyde, all nuclei were stained with Hoechst, and the cells were visualized under a confocal microscope (FV-1000; Olympus Corporation).

### Gene expression

Reverse transcription of RNA to cDNA was performed using the High-Capacity cDNA Reverse Transcription Kit (Applied Biosystems, USA) under the following thermal cycler conditions: 25 °C for 10 min, 37 °C for 120 min, and 4 °C for 5 min. A total of 100 ng of cDNA from each sample was used for subsequent analysis. Quantitative PCR (qPCR) was conducted in a 15 µL reaction mixture containing 7.5 µL of PowerUp SYBR Green Master Mix (Applied Biosystems, USA), 5 µL of cDNA, 1 µL of RNase-free water, and 0.75 µL each of 10 µM forward and reverse primers (Genomed, Poland). Reactions were run on a QuantStudio 5 thermal cycler (Thermo Fisher Scientific, USA) using the following cycling conditions: 95 °C for 10 min, followed by 40 cycles of 95 °C for 15 s and 60 °C for 60 s. Each group was analyzed in quadruplicate. Relative gene expression was calculated using the 2^−ΔΔCT method, with normalization to RPL13A as the reference gene. The primer sequences (Table S2) and qPCR results (Figure S9) are provided in the Supplementary Information. “.

### Statistical analysis

Quantitative results were analyzed using GraphPad Prism (GraphPad Software, San Diego, CA, USA) by performing standard one- or two-way ANOVA. Cytotoxicity assays, lipid peroxidation and cellular membrane potential were analyzed by standard one-way ANOVA with Duncan’s post hoc test, with groups that differed significantly (*p* < 0.05) from the control group marked with [*]. For data from hydrodynamic diameter measurements, angiogenesis and migration assays, ROS production and NF-κB activation results, as well as for gene expression evaluation Tukey’s post hoc tests were conducted. The results are presented as means with standard deviations. Differences between groups are marked with different letters above the bars and considered significant (*p* < 0.05).

## Results and discussion

### Physicochemical differences in Raw and modified NDs

**Table 1:** Positions of particular bands from Raman spectroscopy.

Nanoparticles	Band	Band location [$\:{\mathbf{c}\mathbf{m}}^{-1}]$
ND-R	Diamond	1324
	NCD band	1410
	G band	1590
ND-M1	Diamond	1324
	NCD band	1410
	G band	1590
ND-M2	Diamond	1329
	NCD band	1438
	G band	1575
ND-M3	Diamond	1321
	NCD band	1430
	G band	1575

**Fig. 1:**
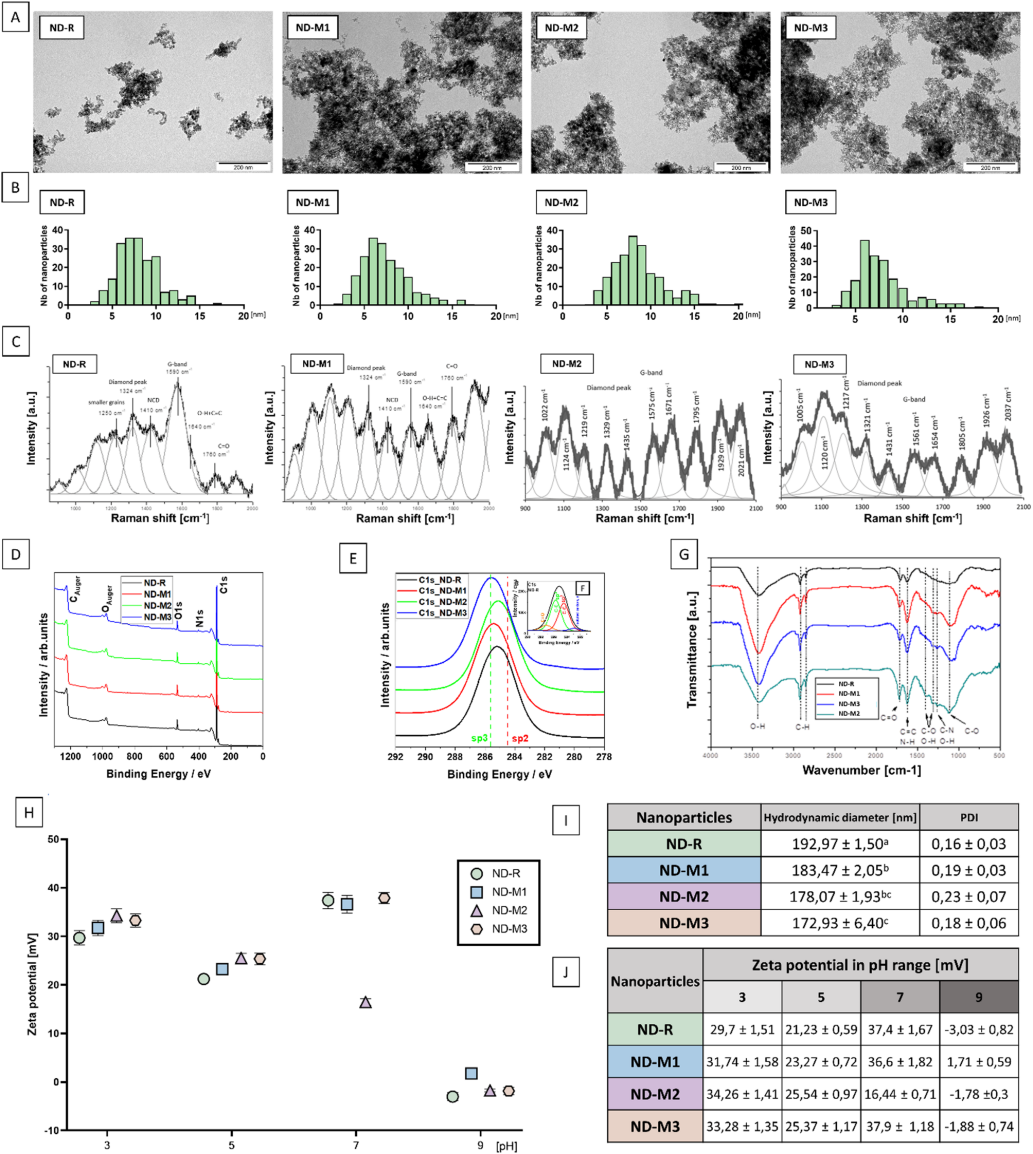
Surface modification resulted in a shift in sp^3^/sp^2^ carbon ratio. Physicochemical characterization of diamond nanoparticles: TEM images (**A**) and calculated size distribution histograms (**B**). Raman (**C**) analysis exposes peaks typical for diamond nanostructures accompanied with “D” and “G” peaks referred to graphite-like carbon. The XPS wide scans (D) and high resolution spectra of C1s carbon for unmodified (ND-R) and modified diamond nanoparticles (ND-M1, ND-M2, ND-M3) (**E**). The inset shows an example of the carbon C1 spectrum after deconvolution procedure for sample ND-R (**F**). FTIR (**G**) examination of surface group composition. Zeta potential of nanoparticle hydrocolloids measured by laser Doppler electrophoresis in the pH range of 3 to 9 (**H**); mean values of hydrodynamic diameter measured by dynamic light scattering (statistically significant differences are indicated by superscripted letters; *p <* 0,05; one-way ANOVA with Tuckey post hoc); polydispersity index (PDI) (**I**), and summary of Zeta potential (**J**) measured in triplicate.

The basic physicochemical characterization demonstrated a high similarity between the studied NDs. TEM images (Fig. 1a) revealed no differences in the morphology of the nanoparticles. Analysis of the size distribution histograms (Fig. 1b) confirmed that the nanoparticles had an average size between 5 and 10 nm. The hydrodynamic diameter of the ND agglomerates in ultrapure water was comparable for all the nanoparticles, with the highest value of 192.97 ± 1.5 nm describing nonfunctionalized NDs, and the lowest value of 172.93 ± 6.4 nm for ND-M3 (Fig. 1i). The samples were characterized by good dispersity, with a PDI (*polydispersity index*) below 0.3 described as acceptable in the nanoparticle-based drug delivery field^20^. ND-M2 showed the greatest heterogeneity with a PDI value of 0.23 ± 0.07, and ND-R had the lowest PDI of 0.16 ± 0.03.

All nanoparticles were characterized by a positive zeta potential in the pH range of 3–7 with the isoelectric point oscillating around slightly basic pH (Fig. 1j). At neutral pH, pristine ND-R along with NDs modified with oxygen-containing groups—NDM1 and ND-M3—exhibited good stability in aqueous solution, with results above the cutoff value of ± 30 mV^21^. ND-M2 had a zeta potential value of 16.4 mV, which indicated its lower stability in physiological pH. The positive surface charge, due to increased electrostatic interactions with proteins, can affect the formation of the protein corona and its remodeling^22^. Although the tendency for aggregation and the graphitic surface content are clearly documented to determine the binding affinity and the activity of NDs in general, the surface charge itself might be a factor that is much more dependent on the environment^23^. Zeta potential can affect nanoparticle–cell interactions, inflammatory response, oxidative stress, and viability, but these outcomes are closely connected to the cell type tested and other factors^20,24^.

In the ND Raman spectra (Fig. 1c; Table 1), typical sharp diamond peaks were noted at a wavelength of 1333 $\:{\text{c}\text{m}}^{-1}$^25^. In the tested samples, an extended signal was present in the range of 1321–1329 $\:{\text{c}\text{m}}^{-1}$, associated with the presence of smaller crystallites^25^. The slight shoulder at around 1250 $\:{\text{c}\text{m}}^{-1}$ observed for the ND-R can also be attributed to the presence of the finest grains, which is described by the phonon confinement model^26–28^. The size distribution obtained from the Raman spectra describes the size of the scattering domain rather than the particle itself, but in the case of NDs, it can be compared to the crystallite size of a diamond core^29^. The second proposed source of this arm, suggested by Prawer et al.,^30^ is the presence of $\:{sp}^{3}$ disordered carbon. Korepanov et al. (2017) assigns to this structure broad bands from a wider range, namely 1100–1350 $\:{\text{c}\text{m}}^{-1}$^25^. Bands around 1430–1438 $\:{\text{c}\text{m}}^{-1}\:$are characterized as “D bands” and are related to higher sp^2^ carbon content^27^. Peaks at 1150 $\:{\text{c}\text{m}}^{-1}\:$are typically attributed to nanocrystalline diamond (NCD) and trans-polyacetylene^31^. Features between 1575 and 1590 $\:{\text{c}\text{m}}^{-1}\:$called “G bands,” which prevail in ND-M2 and ND-R, indicate a higher content of graphitic layer in these samples^28^. Overlapping signals within the range of 1600–1800 $\:{\text{c}\text{m}}^{-1}\:$originate from surface functional groups, where signals around 1760 $\:{\text{c}\text{m}}^{-1}$ are from C = O. Features around 1640 $\:{\text{c}\text{m}}^{-1}$ in the nonmodified ND sample are most likely related to water molecules adsorbed on the surface^25,28^.

XPS measurements show clear signals for carbon C1s, oxygen O1s, and nitrogen N1s in individual samples (Fig. 1d). Detailed analysis of the high-resolution spectra allowed subtle differences to be demonstrated among differently modified samples (Fig. 1e and f – inset). These changes are presented in Table 2, which shows the variation in the elemental composition following successive surface treatments of the nanopowder. Additionally, the positions of characteristic C–C bonds with sp³ and sp² hybridization are indicated in Fig. 1d, based on the deconvolution of the C1s peak at binding energies around 285.6 eV and 284.5 eV, respectively (see inset in Fig. 1e). The most characteristic change was related to the change in the sp^3^ to sp^2^ carbon ratio, indicating successful purification of ND-M1, ND-M2, and ND-M3 compared to pristine ND-R^32^. The lowest sp^2^ carbon content was observed on the surface of the ND-M3 sample, which corresponds to the Raman spectra results.

**Table 2:** Changes in the concentrations of carbon, oxygen, and nitrogen in the nds, and the corresponding sp³/sp² carbon ratio determined from XPS analysis.

Sample	sp^3^/sp^2^ (~ 285.6 eV / 284.5 eV)	N1s At.%	C1s At.%	O1s At.%
**ND-R**	1.11	1.5	94.6	3.9
**ND-M1**	1.34	1.4	94.0	4.1
**ND-M2**	1.61	1.7	93.3	4.5
**ND-M3**	1.85	1.4	93.2	4.7

*Additionally, Cl at the level of 0.5–0.6 At.% was detected in functionalized samples.

An FT-IR peak detected around 3000 $\:{\text{c}\text{m}}^{-1}$ and 3700 $\:{\text{c}\text{m}}^{-1}$ (Fig. 1g), which is more pronounced in ND-M1 and ND-M3 samples, is correlated mainly with hydroxyl groups (-OH) and water^33^. The broad band between 3300 $\:{\text{c}\text{m}}^{-1}\:$and 3400 $\:{\text{c}\text{m}}^{-1}\:$ in ND-M2 could originate from N-H stretching vibrations^33,34^. Smaller features from 2850 $\:{\text{c}\text{m}}^{-1}$ to nearly 3000 $\:{\text{c}\text{m}}^{-1}$ can be attributed to the C-H stretch^33^. The peak at 1720 $\:{\text{c}\text{m}}^{-1}\:$can be assigned to C = O stretching vibrations of carboxyl groups of ND-M3, while the neighboring peak at 1615 $\:{\text{c}\text{m}}^{-1}$ is due to the aromatic (sp^2^ vibrational) C = C bonds of graphitic carbon or N-H bending vibration of -NH₂^33,35^. The peaks at 1400 $\:{\text{c}\text{m}}^{-1}$ and 1100 $\:{\text{c}\text{m}}^{-1}$ are severally attributed to the stretching vibrations of C–OH and coupled C–C/C–O and C–O stretching vibrations^33,36^. Fourier spectra revealed a higher content of oxygen-containing groups in ND-M1 and ND-M3. Moreover, the ND-M2 sample demonstrated features typical of N-H vibrations or graphitic sp^2^ carbon, which was also observed in Raman spectra analysis^32^.

### NDs’ direct cytotoxicity depends on the cell type

**Fig. 2:**
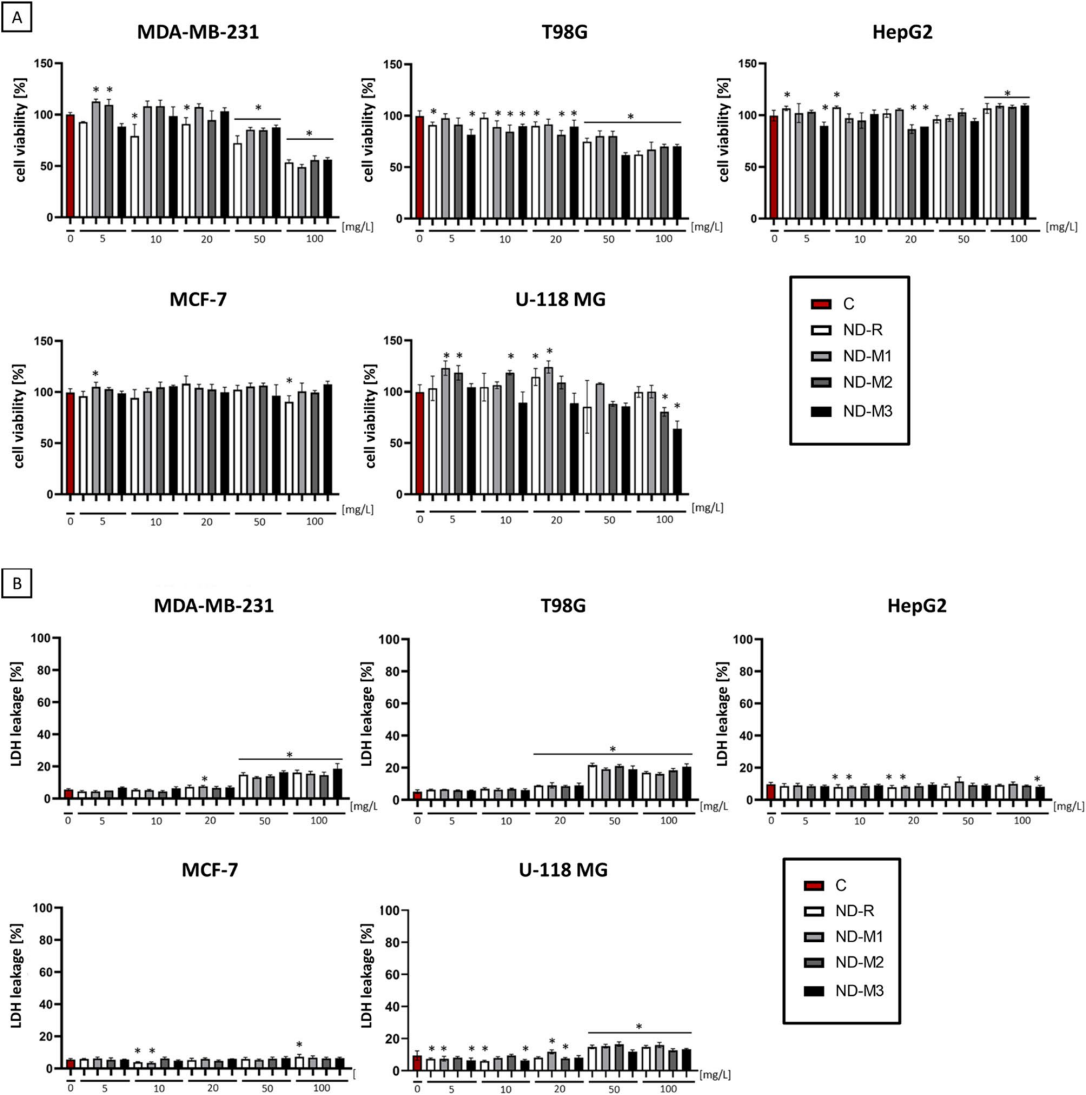
NDs impair the metabolic activity and membrane integrity of MDA-MB-231 and two glioblastoma cell lines, T98G and U118 MG, in a concentration-dependent manner but are nontoxic to HepG2 and MCF-7 cells. The metabolic activity results were established by XTT assay (**A**), while the LDH leakage assay was used for cell membrane integrity evaluation (**B**). Assays were conducted on MDA-MB-231 and MCF-7 breast cancer cells, T98G and U118 glioblastoma cells, and HepG2 hepatocellular carcinoma tumor cells after incubation for 24 h with ND-R, ND-M1, ND-M2, and ND-M3 hydrocolloids at concentrations of 5, 10, 20, 50, and 100 mg/L. XTT results presented as a percentage of the untreated control group. LDH leakage percentage compared to positive control (lysed cells). [*] above the bars indicates a significant difference (*p* < 0.05, two-way ANOVA with Duncan’s post hoc test; *n* = 3) from the control group (“C”).

Among carbon nanoparticles, NDs are considered the most biocompatible^37^. However, as they are intensively internalized by various cell types, they can modulate intracellular signaling pathways and influence cell metabolism, proliferation, and redox potential^38,39^. Regardless of functionalization or concentration, the NDs used had no substantial effect on the viability of the HepG2 and MCF-7 cell lines (Fig. 2). Evaluation of cell membrane integrity revealed that, similarly to the XTT results, neither HepG2 nor MCF-7 cells showed any vulnerability to the presence of NDs even at the highest concentration. These findings are consistent with previous works on hepatocellular carcinoma demonstrating that NDs do not directly affect viability or metabolic activity even at a concentration of 1 mg/mL^40^. Paget et al. also reported no cytotoxic effect of ND-COOH with different diameters of around 20 nm and 100 nm in liver, lung, kidney, and intestine cell lines^41^. It has also been confirmed that low concentrations are biocompatible with MCF-7^42,43^. Here, we reported no toxic effects at concentrations of up to 100 mg/L.

In contrast, a cytotoxic effect depending on the concentration and surface modification was observed in MDA-MB-231 breast cancer cells (Fig. 2a). A decrease in MDA-MB-231 viability after ND-R treatment was already visible at a concentration of 10 mg/L, whereas a reduction in viability by modified NDs was observed from a concentration of 50 mg/L. ND-M1, ND-M2, and ND-M3 at 50 mg/L decreased the metabolic activity of MDA-MB-231 by 15–18%, while ND-R caused a 28% decline. Treatment with all NDs at 100 mg/L resulted in a nearly twofold decrease in viability compared to the control. The values of free LDH obtained for all the NDs at both 50 mg/L and 100 mg/L differed from the negative control by an average of 10%, with a maximum 13% increase for ND-M3 at 100 mg/L. In this case, surface modification improved the cytocompatibility of NDs at low concentrations, although changes in surface composition did not influence the interaction with cell membrane. A similar tendency for low concentrations of ND-M1 and ND-M2 was observed in the viability of the U118 MG cell line. NDs concentrations of up to 20 mg/L were indifferent to cells or enhanced their viability (ND-M1 5 mg/L by 23%; NDM1 20 mg/L by 24%; NDM2 5 mg/L and 10 mg/L by 18%). The metabolic activity of U-118 MG significantly decreased after treatment only with the highest concentrations of ND-M2 and ND-M3. In addition, the increase in LDH outflow was not greater than 7% in relation to spontaneous LDH leakage. Treatment with ND-M3 caused a more pronounced decrease in viability at 50 mg/L (38%) compared to the results for the other modified NDs, which oscillated between 20% and 25%. Treatment with nanoparticles at concentrations of 50 mg/L and 100 mg/L resulted in an increase in LDH outflow of 11–16%, respectively.

Other studies demonstrated a similar decrease in the viability of U87 glioblastoma cells to that presented for T98G cells after treatment with nonmodified NDs, but at concentrations starting from 100 mg/L, and a slight decrease in the viability of the less invasive U-118 MG cell line, which corresponds to the results presented in this work^44^.

Nanoparticles interact with the cell membrane in a manner dependent on their size, shape, and surface chemistry^37,45^. Therefore, evaluating membrane integrity is an important factor in the comparative analysis of different types of NDs. The above results indicate, as was also presented in previous works, that NDs of up to 100 mg/L effectively enter cells without markedly impairing the cell membrane and affect metabolic activity in a cell-specific manner^38,46^. Aggregate overload was observed only in cells treated with a higher concentration of NDs (see Supplementary Figures S1, S3, and S4), leading to cell swelling and ultimately some minor cell membrane disruptions (Fig. 2). MCF-7 and HepG2 cells exhibit an epithelial phenotype and strong adhesion, form tight intercellular junctions, and grow in cobblestone-like patches, while MDA-MB-231, T98G, and U-118 MG are characterized by a more migrative, mesenchymal phenotype. The different cell characteristics could have been the cause of the better availability of the nanoparticles for MDA-MB-231 cells, which led to cell shrinkage and overloading with the NDs (see Figure S1). In contrast, similar morphology disruptions were not observed in the case of the second line of breast cancer—MCF-7—which also corresponds to the cytotoxicity results (see Supplementary Figure S2; Fig. 2). Overloading with diamond nanoparticle agglomerates around the nucleus as well as thin and elongated protrusions were also visible in glioblastoma cell lines (see Supplementary Figure S3; Figure S4). HepG2 cells treated with higher ND concentration grew in more relaxed, expanded clusters (see Supplementary Figure S5).

This enhanced endocytosis may be due to the stronger metastatic potential and mesenchymal phenotype of MDA-MB-231 cells and glioma cell lines compared to MCF-7 and HepG2 cells^47^. Nevertheless, the effect was not determined by surface modification.

Nanoparticles demonstrated no to moderate direct toxicity in singular groups of up to 50 mg/L. Our goal was to evaluate the possibility of regulating the proangiogenic features of tumor cells with NDs without causing direct apoptosis. Based on the results obtained, a concentration of 50 mg/L was selected for further experiments.

### Depending on the surface chemistry and cell type, NDs can indirectly inhibit or promote tube formation

**Fig. 3:**
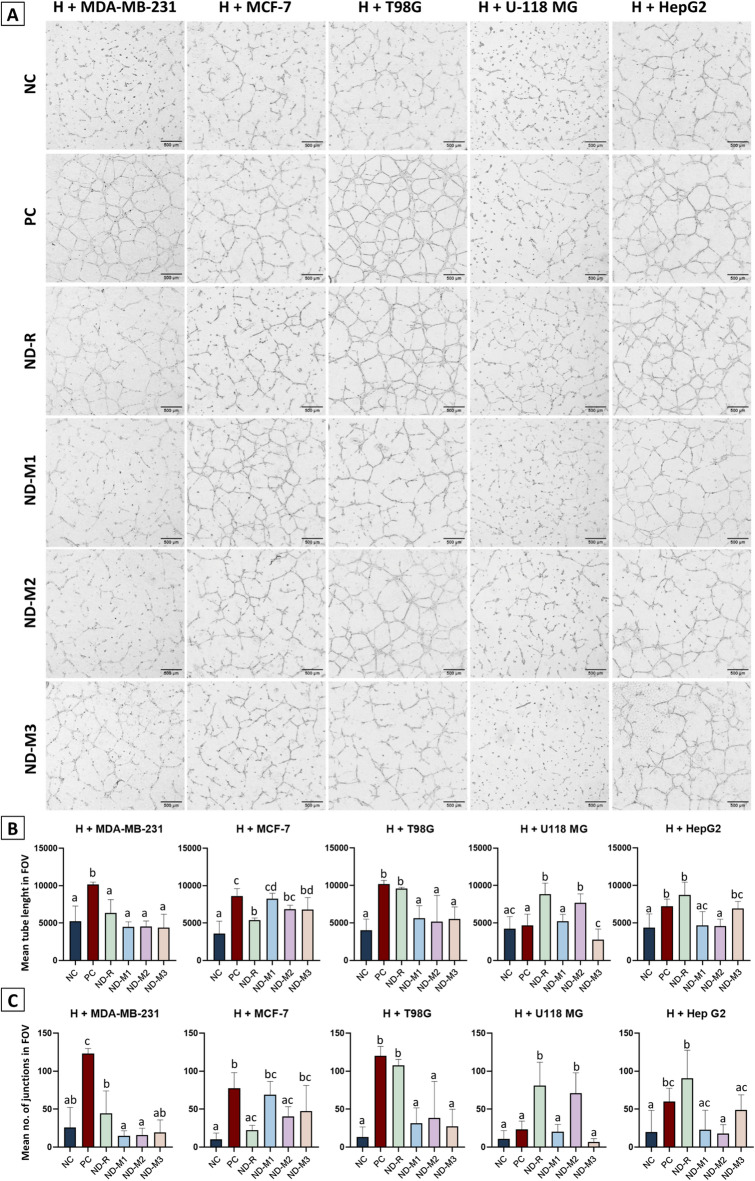
The influence of NDs on the proangiogenic potential of tumor cells is cell type-specific and determined by surface chemistry. Images of HUVECs’ tubular structures after 7 h of incubation on the extracellular matrix, which was preceded by 24 h of incubation with the respective CM (**A**); scale bar 50 μm. The negative control (“NC”) contained Basal ECGM 1:1 EMEM 0% FBS; the positive control (“PC”) contained Basal ECGM 1:1 CM from untreated tumor cells (MDA-MB-231, MCF-7, T98G, U-118 MG, and HepG2); the tested combinations included CM derived from five tumor cell lines treated with ND-R, ND-M1, ND-M2, and ND-M3 at 50 mg/L. The mean tube length (**B**) and mean number of junctions (**C**) in the FOV (field of view) were calculated by ImageJ software. The results are presented as the mean value with standard deviation; different letters above the bars indicate significant differences (*p* < 0.5, one-way ANOVA with Tukey’s post hoc test).

A tube formation assay was performed to investigate the ability of individual tumor cell types to promote tube formation by HUVECs. We employed an indirect co-culture model to better reflect the characteristics of endothelial cells in the tumor microenvironment, as the phenotype, morphology, and protein profile of endothelial cells change depending on the type of medium conditioning^17^.

Furthermore, we assessed the indirect influence of NDs on the regulation of this process and compared the effects of modified and raw NDs. Images of tubes formed within a 7-hour assay revealed that MDA-MB-231 CM and T98G CM exhibited greater proangiogenic potential than conditioned media from other breast cancer (MCF-7) and glioblastoma (U-118 MG) cells (Fig. 3a). As predicted, treatment with raw ND-Rs in both types of breast cancer CM-conditioned HUVECs significantly reduced the number of tubular structures, but interestingly did not cause any change in T98G- and HepG2 CM-conditioned HUVECs (Fig. 3). Moreover, it had a positive effect on tube formation in HUVECs conditioned with U-118 MG CM. Regardless of the type of functionalization, the modified NDs diminished tube length (Fig. 3b) and the number of junctions (Fig. 3c) in both MDA-MB-231 CM- and T98G CM-conditioned HUVECs. Tubes formed by HepG2 CM-conditioned HUVECs were altered by ND-M1 and ND-M2, but not ND-M3. In U-118 MG CM–conditioned HUVECs, ND-M3 caused a significant decrease in tube length but not in the number of junctions, while ND-M2 had similar properties to nonfunctionalized NDs.

A decrease in the number of cell branches and junctions may result in lower stability and leakiness of newly formed vessels. Setyawati et al. demonstrated changes in vascular permeability caused by the presence of NDs with different functional groups. Amino-functionalized NDs caused an increase in vascular leakiness associated with the production of ROS and activation of the extracellular signal-regulated kinase (ERK) pathway, which resulted in better penetration of the cytostatic drug^48^. Enhanced ROS production is often linked with negatively charged nanoparticles^49^. All nanoparticles used in the Setyawati study had negative zeta potential in aqueous solution, whereas in our study, all of them were positively charged. Modified NDs inhibited tube formation, but without pronounced differences among groups. The only exception was U-118 MG CM, where ND-M3 had the strongest inhibitory effect and ND-M2 improved tube formation, which can be correlated with other studies reporting that aminated NDs are nontoxic to neural cells^50,51^. In studies on the effect of different positively charged carbon allotrope nanomaterials on glioblastoma angiogenesis, graphene oxide and graphite similarly did not affect the proangiogenic activity of U-118 MG but decreased tube formation in HUVECs cocultured with U87 cells through the downregulation of proteins connected to the NF-κB signaling pathway^52^. This phenomenon proves the strong cell type and ND surface chemistry dependence in the mechanism of interaction.

### Higher sp^3^ carbon content on surface of NDs inhibits endothelial migration regardless of the CM origin

**Fig. 4:**
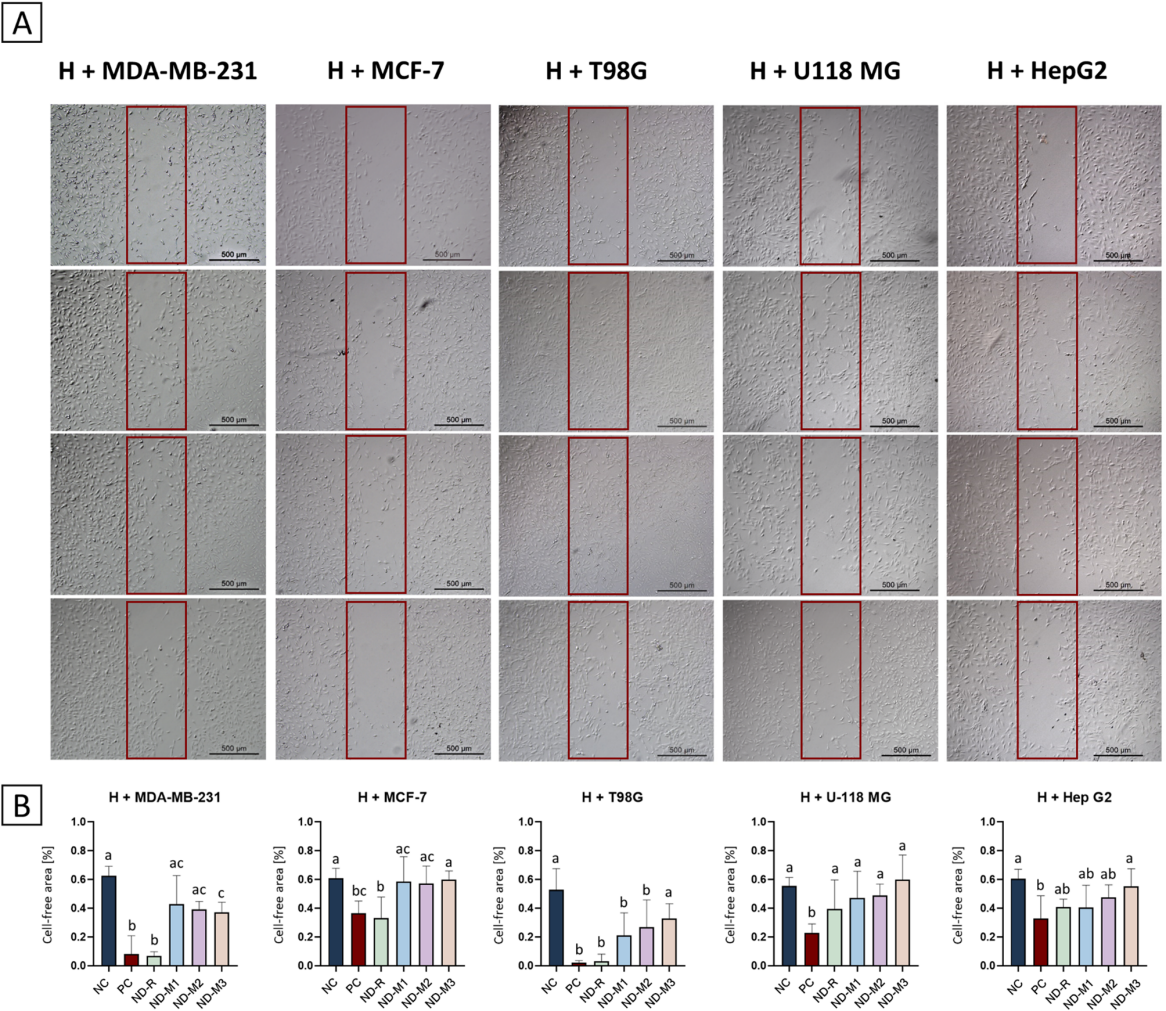
Surface-modified NDs restrain the promigratory effect of tumor cells in indirect coculture. Effects of CM derived from MDA-MB-231, MCF-7, T98G, U-118 MG, and HepG2 cells on the migration of HUVECs (“H”). Images of migrating HUVECs after 24 h incubation (**A**) with ECGM 1:1 EMEM 0% FBS as a negative control (“NC”), ECGM 1:1 CM from untreated tumor cells as a positive control (“PC”), ECGM 1:1 CM from tumor cells treated with nonfunctionalized NDs at a concentration of 50 mg/L (“ND”), and ECGM 1:1 CM from tumor cells treated with ND-M3 at a concentration of 50 mg/L (“ND-M3”) acquired by inverted microscopy; scale bar = 500 μm; free space remaining immediately after removing the insert is outlined in red. Quantified representations of the obtained results as a percentage of unoccupied space relative to the beginning of the test and to the NC (**B**). The letters above the bars indicate significant differences (*p* < 0.05, one-way ANOVA with Tukey’s post hoc test).

The results obtained from the migration assay demonstrated strong stimulation of endothelial cells by MDA-MB-231 and T98G CM (Fig. 4a), which corresponds to their effects on tubular structure formation. The most pronounced decrease in migration was observed for ND-M3 in HUVECs conditioned with two glioblastoma-derived CMs and for ND-M1 in HUVECs with MDA-MB-231 CM. In the above groups, the percentage difference in the cell-free area compared to the positive control exceeded 30%. Although there were marginal differences between the modified NDs, only the ND-M3 treatment demonstrated a statistically significant difference from the positive control in every conditioning variant (Fig. 4b). This phenomenon could have a basis in what was presented previously, that NDs with -COOH functional groups tend to inhibit tumor cell migration and metastasis by increasing the epithelial E-cadherin to mesenchymal N-cadherin ratio, lowering the promigratory protein level, and improving adhesion^53^ Additionally, Chen et al. revealed that ND-COOH at concentrations above 50 mg/L can, without affecting viability, considerably inhibit HepG2 cell migration and downregulate the expression of epithelial–mesenchymal transition-related genes, which can further change the effect of tumor cell stimulation of endothelial cells^54^. Moreover, Guo et al. reported enhanced migration and cancer-like characteristics in HUVECs co-cultured with HeLa cells. However, treatment with carboxylated nanodiamonds (NDs) in a microfluidic platform inhibited both endothelial cell migration and proliferation. Notably, this study did not include a comparison with non-functionalized nanoparticles or NDs with alternative surface chemistries, limiting conclusions about the specific role of surface modification^55^. Eventually, this functionalization is strictly connected with purification of the NDs’ surface. As the NDs used in this research exhibited similar indirect effects on cell migration, while they did not demonstrate the great advantage of carboxyl surface groups, another explanation could be that the sp^3^/sp^2^ carbon ratio is a structural parameter determining this cellular response to NDs.

**Fig. 5:**
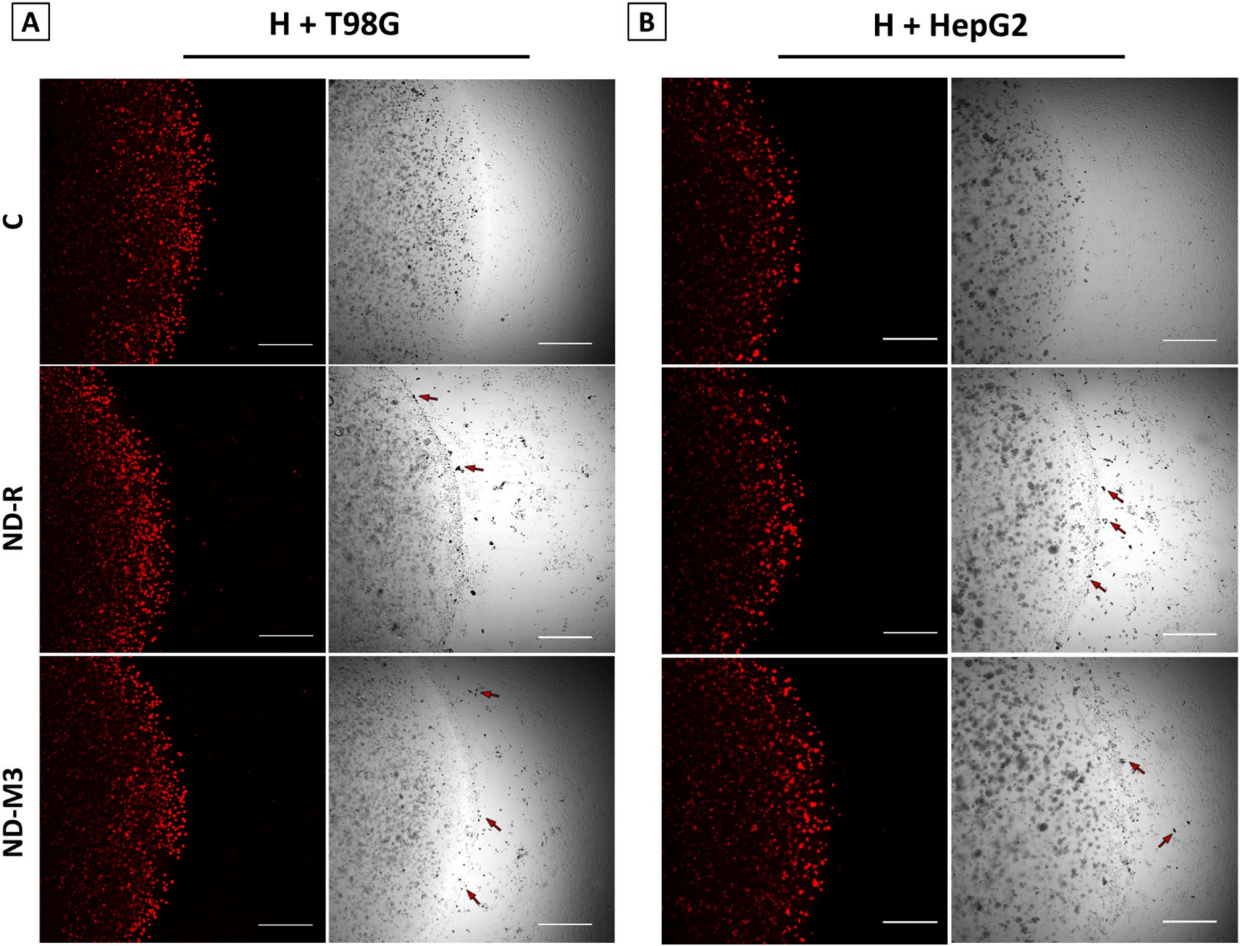
NDs affect the number of HUVECs in the T98G bioprinted droplet border area but not in the HepG2 coculture. Hybrid tumor angiogenesis model consisting of 3D-printed droplets with encapsulated T98G (**A**) and HepG2 (**B**) cells and HUVECs in 2D culture. Tumor cells were stained with Cell Tracker DeepRed (Thermo Fisher Scientific). ND aggregates are marked with arrows. The constructs were treated with 50 mg/L ND-R or ND-M3 hydrocolloids.

In a bioprinted hybrid model of the tumor microenvironment, after 13 days of total incubation, the sphere structure remained stable. Singular aggregates of NDs were visible at the edges of the structure. HUVECs maintained in 2D culture surrounding 3D droplets grew evenly in the untreated groups, yet after both ND-R and modified ND-M3 treatment, a cell-free area was formed around the bioprinted tumor spheroids. This effect was observed only for T98G (Fig. 5a). The growth pattern of HUVECs cocultured with HepG2 cells was unchanged after nanoparticle treatment.

### The sp^3^/sp^2^ carbon ratio determines the cellular redox state

**Fig. 6:**
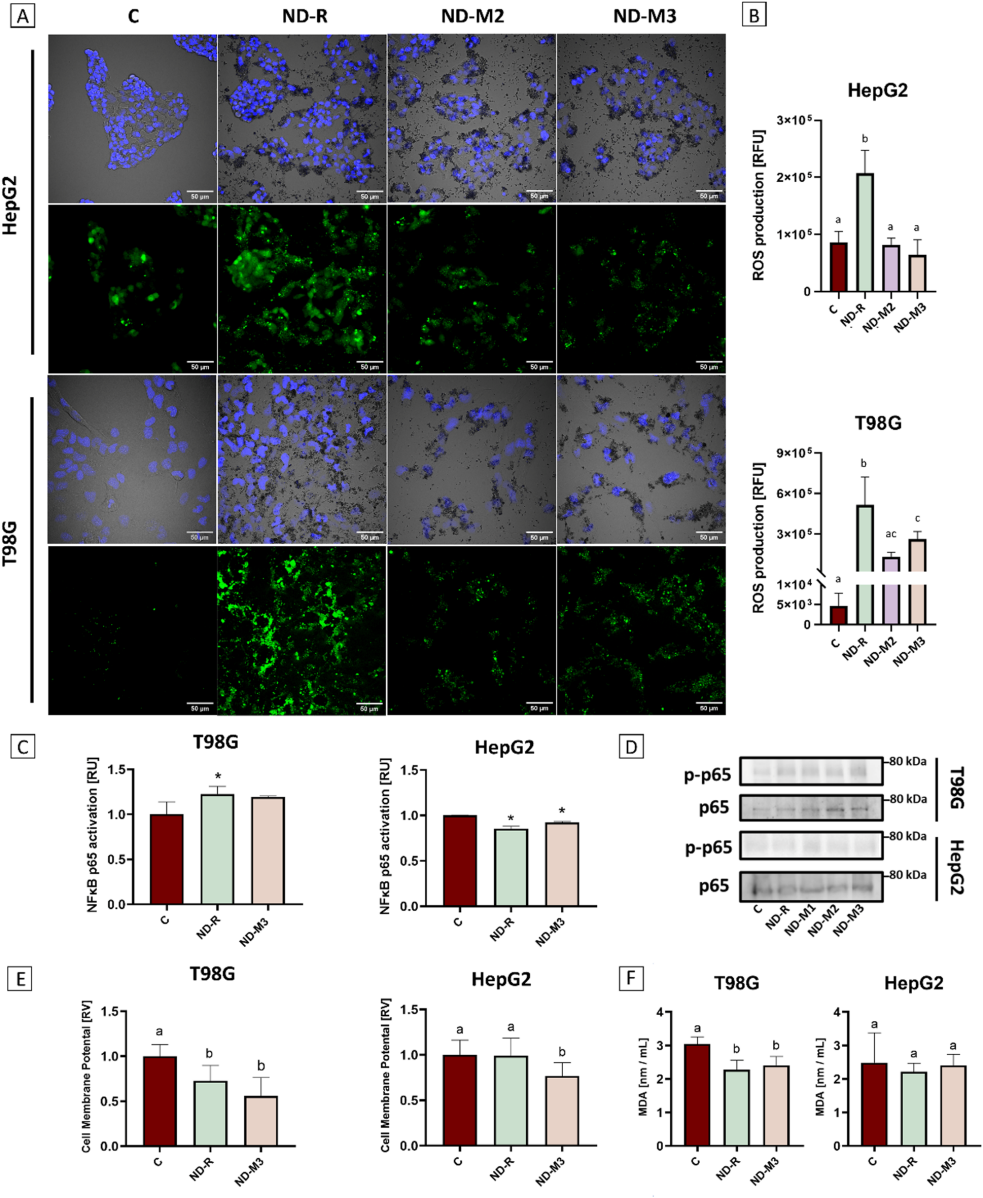
A higher sp^3^ carbon ratio decreases total ROS production in tumor cell lines and affects the NF-kB p65 activation in a cell type-dependent manner. Total ROS production in HepG2 and T98G cells after 24 h incubation with ND-R, ND-M2, and ND-M3 at 50 mg/L (**A**). ROS were stained with CM-H2DCFDA (Thermo Fisher) and visualized with confocal microscopy. Fluorescence intensity was measured using ImageJ software (**B**); RFU – relative fluorescence units. The results of an ELISA-based assay targeting the NF-kB p65 subunit activation state (Active Motif) performed in triplicate on T98G and HepG2 cells after 24 h incubation with ND-R and ND-M3 at 50 mg/L (**C**). Groups significantly different from the control are marked with asterisks [*]. RU – relative units. Phosphorylated and total p65 detected by western blot (**D**). Membranes from T98G and HepG2 cell lysates pretreated for 24 h with 50 mg/L ND-R, ND-M1, ND-M2, or ND-M3. “C” indicates the untreated control group.

The total amount of ROS in T98G and HepG2 cells was measured after 24 h exposition on nanoparticles. In both cell lines, raw NDs induced intensive oxidative stress. In HepG2 cells, ROS production was approximately twice as great as that in the control group, whereas treatment with modified ND-M2 and ND-M3 left ROS at the control group level (Fig. 6a and b). In T98G, ND-R increased ROS production a hundredfold. Comparing modified NDs to raw NDs, ND-M3 reduced ROS levels by 50%, while ND-M2 decreased ROS levels by two thirds. Furthermore, neither of the utilized NDs variants induced lipid peroxidation (Fig. 6f). Regardless of the cell line examined, cells treated with nanoparticles for 24 h exhibited a slight reduction in MDA levels compared to the untreated control. Lee et al. reported differences in intrinsic ROS generation associated with only the sp^3^/sp^2^ carbon ratio. Among seven nanodiamonds, all of which were negatively charged, the nanoparticles with the highest sp^3^ carbon content showed the lowest ROS levels. Moreover, most of the studied nanodiamonds induced inflammatory response observed as elevated production of proinflammatory interleukins and increased neutrophil numbers in rat bronchoalveolar lavage fluid, while none of those outcomes were detected for NDs with the highest sp^3^ carbon^15^. In many studies, NDs have demonstrated anti-oxidative and enzyme-mimetic activity, protecting cells from oxidative stress^56,57^. However, it has been suggested that the availability of oxygen-containing surface groups accompanied by the reduction of the outer amorphous carbon layer is essential for this activity^56^. Moreover, a high level of ROS typically leads to lipid peroxidation. Nevertheless, there is existing evidence that other carbon-based nanomaterials, such as fluorescent nanodiamonds, can activate the cellular antioxidant system without triggering lipid peroxidation, thereby helping to mitigate cell damage caused by free radicals^57^. Maintaining this balance is particularly important in tumor therapies, as oxidative stress can either promote tumor growth or enhance therapeutic efficacy^58^. ROS levels can modulate the activity of the NF-kB factor complex, which plays a crucial role in tumor development and angiogenesis. NF-kB is connected with the inflammatory response and secretion of proangiogenic cytokines and is often activated under stress conditions. A higher phosphorylation state of the NF-kB p65 subunit, marked in various cancers, often indicates the positive regulation of IL-6, IL-8, and VEGF-dependent angiogenesis^59,60^. In HepG2 cells, incubation with both NDs caused a minor decrease in the phosphorylation of p65 (Fig. 6c), without affecting the total protein level according to western blot analysis (Fig. 6d). Although the results for T98G showed overall low levels of phosphorylated p65, they confirmed a slight increase in phosphorylated p65 after ND treatment^53^. Based on our results and those of other studies, NDs with a higher sp^3^ carbon content influence the cellular redox state, though they exhibit greater tolerance over time compared to raw NDs and contribute to the silencing of inflammation with suppressed NF-kB signaling^15^. Notably, the dynamics of the cellular response is highly dependent on the cellular phenotype, as the downregulation of NF-kB p65 was detected only in epithelial HepG2 cells.

An additional factor distinguishing cell phenotypes is the state of cellular membrane polarization. Tumor cells are generally characterized by a higher membrane potential compared to non-tumor cells^61,62^. NDs may mitigate the invasive properties of tumors such as glioblastoma, represented here by T98G cells, through interactions with membrane-bound organelles and ion channels, potentially leading to a reduction in depolarization potential. A significant decrease in membrane potential was observed for both cell lines only in response to ND-M3, suggesting enhanced cellular availability in HepG2 cells, which display a patch-like growth pattern. In contrast, both ND variants caused a reduction in membrane potential in T98G cells.

### Surface-modified NDs downregulate proangiogenic and Proinflammatory proteins secreted by tumor cells

**Fig. 7:**
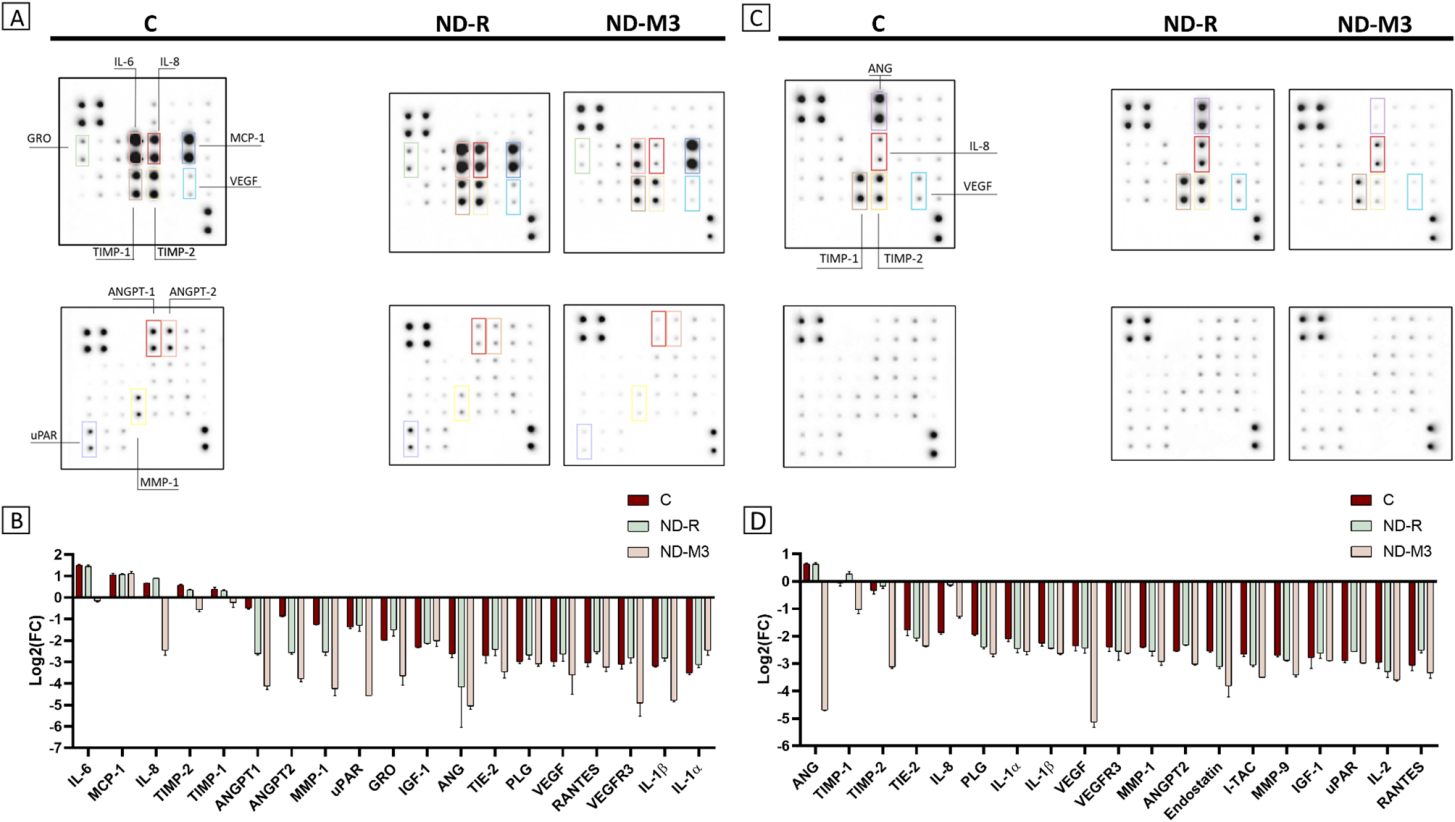
ND surface functionalization led to a significant reduction in the secretion of proangiogenic proteins by T98G glioblastoma cells and HepG2 hepatocellular carcinoma cells. Antibody array allowing for 43-target screening performed on cell culture medium derived from T98G (**A**) and HepG2 (**B**) cells after 24 h incubation with ND-R, with ND-M3 at a concentration of 50 mg/l, and without treatment (control – “C”). The proteins with the most significant changes are highlighted with different colored frames. Localization of the positive controls on the membranes: A1-B2, H7,8. Quantitative representation of results is constituted by Log_2_(FC) (fold change) of protein levels in treated groups compared to the untreated control group (“C”) ordered by their level in the untreated control group (**C**,**D**). The graph demonstrates 19 proteins ranked by their level in the untreated control, which secretion changed after treatment.

To gain deeper insights into the changes in the proangiogenic potential of cancer cells caused by different NDs, we evaluated a secretion panel from T98G and HepG2 cell lines, which represent various phenotypes. The proteins most intensively secreted by T98G cells were monocyte chemoattractant protein-1 (MCP-1), interleukins IL-6 and IL-8, and TIMPs (Fig. 7a and c), while in HepG2 medium, the highest levels were observed for angiogenin (ANG) and TIMPs (Fig. 7b and d). Interestingly, 50 mg/L of modified NDs significantly decreased the production of most of the detected proteins, among which were interleukins IL-6, IL-8, IL-1β, TIMPs, MMPs, ANG, ANGPTs, growth-regulated oncogene (GRO), urokinase type plasminogen activator receptor (uPAR), and others. In contrast, in the group treated with nonfunctionalized NDs, most of those proteins remained at the untreated control level. Moreover, the production of GRO in T98G and IL-8 in HepG2 medium increased, and only ANGPTs and MMP1 levels in T98G-derived medium were lower than those in the untreated control. MCP-1 was detected at the same level in T89G medium in both the treated and untreated groups. Moreover, ND-M1 caused an additional elevation up to three times higher than in the control (see Supplementary Figure S7). Increased secretion of MCP-1 is associated with activity of the transcription factors responsible for inducing the epithelial-mesenchymal transition, like NF-kB^63,64^. In addition to the attraction of monocytes, MCP-1 is associated with the activation of angiogenesis and enhanced invasiveness by paracrine and autocrine stimulation of secretion of cytokines, such as IL-6 or IL-1β, and IL-1α^64,65^. Diamond nanoparticles, as demonstrated previously, can trigger or silence inflammation-related cell responses^66–68^.

According to Zanotto-Filho et al., IL-6 is more responsible for autocrine stimulation of invasiveness, while IL-8 paracrinely promotes angiogenesis^69^. Moreover, it was shown that inhibition of MCP-1 decreases metastasis in breast tumors, but not in liver tumors, indicating that alternative mechanisms are engaged in the promotion of tumor growth^70^. Our research also demonstrated that proangiogenic mechanisms in HepG2 cells do not involve MCP-1 related pathways. The greatest changes in protein level secreted by T98G were observed for proinflammatory IL-6 and IL-8 in the ND-M3-treated group, with no difference in IL-6 and a minor increase of IL-8 in the ND-treated sample compared to the control group. The above-mentioned interleukins in the control HepG2 medium were at a low level, but ND treatment led to the upregulation of both IL-8 and IL-6. McFarland et al. accomplished the highest survival rates for additive therapy in glioblastomas with NF-kB and STAT3 inhibitors, keeping both IL-6 and IL-8 at reduced levels^71^. Lin et al., studying MDA-MB-231 cells in vitro, also reported that combined blocking of IL-6 and IL-8 most effectively inhibited migration and colony formation^72^. The effective downregulation of both interleukins by ND-M3, without pronounced impairment of NF-kB p65 activity and MCP-1 levels, indicates the involvement of ND-M3 in the alternative pathway apart from canonical NF-kB/MCP-1 signaling. This is contrary to the results obtained by Fusco et al. for NDs functionalized with -COOH and -NH₂ groups studied in peripheral blood mononuclear cells (PBMC). Both NDs showed good hemocompatibility, even at high concentrations, but ND-COOH more explicitly activated the immune system by causing intensified production of proinflammatory cytokines (IL-6, IL-10, IL-1β, TNF-α), decreasing the viability of B lymphocytes and monocytes at the highest concentration and with prolonged exposition, as well as increasing monocyte activation markers^68^. In this case, the effect of silencing the inflammation by modified ND-M3 is mostly connected to reduced graphitic layer rather than the presence of functional surface groups. On the other hand, Shawqi et al., presenting the neuroprotective effect of ND in the context of neurodegenerative changes in Alzheimer’s disease, showed a decrease in the amount of NF-kB and IL-6 with a simultaneous increase in phosphorylated STAT3 (pSTAT3)^73^. This divergence again highlights the character of the cellular response’s dependence on cells’ phenotype and tissue specificity.

ANG, which was the most intensively secreted into the HepG2 medium, both in the control and in the ND-treated sample, remained at a constant level. Nonetheless, treatment with ND-M3 resulted in a more than fivefold decrease in ANG level in HepG2-derived medium. Elevated ANG expression is observed in hepatocellular carcinoma and is directly correlated with enhanced tumor vascularization, invasiveness, and drug resistance^74^. Moreover, ANG is involved in pro-survival and pro-invasive NF-kB crosstalk^75^.

ND-M3, but not raw NDs, reduced the VEGF level in HepG2 medium, yet in both cell lines, the control levels of that protein were low, which indicates the predominance of alternative pathways triggering angiogenesis in the above tumors. This finding suggested that ND-M3 may affect VEGF-dependent angiogenesis, while it also has potential as a multi-target agent. TIMPs and MMPs are groups of proteins involved in extracellular matrix (ECM) degradation; they trigger vascular leakiness, facilitating the extravasation of cells. There are many conflicting reports on TIMP-1 and TIMP-2, but their increased expression is often noted in invasive tumors, such as glioblastoma, breast cancer, and hepatocellular carcinoma, especially in the mesenchymal subtype, and is associated with a detrimental prognosis^76^. Our study demonstrates a decrease in secretion of both TIMP-1 and TIMP-2 with an additional threefold decline in collagenase MMP-1 level in T98G medium. This is a desirable effect, which was also observed in patients examined before and after glioma resection, where the daily measured levels of MMP-1 and TIMPS-1,2 decreased concomitantly with an increase in TIMP-4 levels.^77^ While the levels of these proteins in the hepatocellular control cell media were equal to each other and to those in the T98G medium, the HepG2 cells showed greater sensitivity to ND treatment, with a nearly twofold decrease in the production of TIMP-1 and an approximately threefold decrease in the TIMP-2 level. A significant reduction in the level of TIMP-2 may correlate with a worse prognosis but only with the simultaneous strong expression of MMPs, particularly MMP-2.^78^

Angiopoietins (ANGPTs), which are ligands for tyrosine kinase receptor 2 (Tie-2), determine the stabilization of newly formed vessels. They are usually overexpressed in glioblastoma, and as a consequence, the resulting vessels are characterized by an immature structure, insufficient support by pericytes, and leakiness. This is mainly due to the increased production of ANGPT-2, which competes with ANGPT-1, causes vascular destabilization, and prevents the silencing of endothelial cells.^79^ Blocking ANGPT-2 impedes sprouting angiogenesis and tumor growth.^79^ Our results showed equal downregulation of both angiopoietins in the T98G cell line, with a more pronounced effect after modified ND-M3 treatment. In the case of HepG2 medium, only ANGPT-2 was detectable, but its decrease was accompanied by decreased levels of its receptor Tie-2 and VEGF. An additional proposed mechanism of action of NDs is that the remaining ND aggregates that did not enter the cells bound some of the secreted cytokines^66^. Similarly, the modified ND-M3 used in our research, due to its higher purity and colloidal stability, exhibited greater affinity for cytokines in the medium, which resulted in a decrease in their concentration after removing the nanoparticles from CM compared to that in the medium with raw NDs.

To further link the physiological response of endothelial cells with changes in the cancer cell secretome, we analyzed the expression of three genes essential for endothelial regulation: *KDR* (kinase insert domain receptor) encoding VEGFR2, *NOS3* (nitric oxide synthase 3) and *CAV1* (caveolin-1).

As expected, in HUVECs incubated with CM from T98G cells, a significant decrease in the expression of *KDR* (*Flk-1*,* VEGFR2*), as well as the *CAV1*, was observed only in the ND-M3 group.^80^ In contrast, incubation with CM from T98G cells treated with ND-R resulted in no significant change in the relative expression of these genes compared to the control group. A similar trend was observed in *NOS3* expression, a gene involved in regulating endothelial cell proliferation, oxidative stress response, VEGF signaling, and cytokine-mediated pathways.^81^ Incubation with CM from MDA-MB-231 cells resulted in significantly higher expression of *KDR* and *CAV1* in the ND-R group, which is consistent with our migration assay results and suggests a pro-migratory effect. Conversely, elevated *NOS3* levels in HUVECs incubated with CM from ND-R–treated HepG2 cells correlated with enhanced tube formation, reflecting *NOS3’*s central role in angiogenesis.^82^ These changes were consistent with the results of the angiogenesis assay, as *NOS3* is primarily associated with tube formation.^83^ Caveolin-1 is known to act as a negative regulator of eNOS, although some studies suggest that this interaction can be silenced under stress conditions, such as *hypertension and* pregnancy—an observation that aligns with our gene expression results.

^84^ Previous studies have shown that diamond nanoparticles can cause vascular impairment in the CAM model, an effect associated with reduced expression of the KDR receptor^11^. In summary, our findings suggest that highly purified diamond nanoparticles indirectly downregulate genes involved in endothelial proliferation, migration, and sprouting. In contrast, nanoparticles with a higher sp²/sp³ carbon ratio may, in some cases, promote indirect activation of endothelial cells by increasing the expression of genes such as *KDR* and *NOS3*, potentially reducing their therapeutic effectiveness. These results further highlight the distinct ways in which diamond nanoparticles regulate intercellular signaling and their specific impact on key processes involved in endothelial development. While we acknowledge the complexity of factors shaping tumor microenvironment modulation, these findings provide a strong foundation for extending the study to advanced 3D models that better mimic in vivo tissue conditions. Moreover, our results—demonstrating detailed secretome profiling and cell-type–dependent modulation of angiogenic responses—may support the development of diamond nanoparticle–based therapeutic strategies, particularly in the context of highly invasive tumors. Future research should explore a broader range of surface modification variants, including different functional group densities and surface saturation levels, to better define the relationship between nanoparticle structure and biological activity.

## Conclusions

NDs with a higher sp³/sp² carbon ratio effectively reduce the proangiogenic activity of tumor cells by downregulating key cytokines such as IL-6, IL-8, TIMP-1, TIMP-2, ANG, and ANGPTs. This effect was confirmed in both 2D and 3D models and correlated with the baseline angiogenic potential of each tumor cell line. NDs with lower surface graphitization also reduced oxidative stress and NF-κB activation. Epithelial-like tumor cells (HepG2, MCF-7) were less sensitive to ND exposure than mesenchymal-like cells (MDA-MB-231, T98G). These findings highlight the critical role of core carbon structure in determining ND bioactivity and its relevance for designing nanomaterials targeting the tumor microenvironment.

## Data Availability

The datasets analyzed during the current study are available from the corresponding author on reasonable request.
